# High-Resolution Dipole Remote Detection Logging Based on Optimal Nonlinear Frequency Modulation Excitation

**DOI:** 10.3390/s24196253

**Published:** 2024-09-27

**Authors:** Xueshen Cao, Hao Chen, Chao Li, Yinqiu Zhou, Dehua Chen, Jiaheng Zhao

**Affiliations:** 1 State Key Laboratory of Acoustics, Institute of Acoustics, Chinese Academy of Sciences, Beijing 100190, China; chh@mail.ioa.ac.cn (H.C.); chaoli@mail.ioa.ac.cn (C.L.); zhouyinqiu@mail.ioa.ac.cn (Y.Z.); chendh@mail.ioa.ac.cn (D.C.); zhaojiaheng@mail.ioa.ac.cn (J.Z.); 2 University of Chinese Academy of Sciences, Beijing 100049, China; 3 Beijing Engineering Research Center of Sea Deep Drilling and Exploration, Institute of Acoustics, Chinese Academy of Sciences, Beijing 100190, China

**Keywords:** remote detection, dipole logging, nonlinear frequency modulation, optimization

## Abstract

In order to improve the detection range and imaging resolution of dipole remote detection logging, an optimal nonlinear frequency modulation (ONLFM) excitation method is proposed in this paper. In this method, the optimal waveform model of the sound source is designed with objective functions of SNR and resolution to obtain the highest resolution under the condition of the required SNR. This optimal model is a multi-constraint optimization problem, and the simulated annealing method has been used to solve it. Solving the optimal model can obtain the optimal spectrum, and the ONLFM waveform can be designed by using the stationary phase principle. Compared with the electric pulse sound source used in traditional technology, this ONLFM sound source can improve the energy and extend the frequency band range of the reflected wave, which can provide the higher resolution and SNR. The simulation results show that the ONLFM sound source can effectively improve the SNR and resolution of the reflected wave, and the detection range and imaging resolution of dipole shear wave remote detection will be improved.

## 1. Introduction

Dipole acoustic remote detection logging is an important acoustic logging technology, which can image the anomalous structure outside the well by receiving the reflected wave signal [1]. This technique can increase the detection distance from several meters of traditional acoustic logging to tens of meters and plays an important role in detecting fractures and determining formation structures [2].

Electric pulse is a traditional way to excite the sound source in the dipole remote acoustic logging [3,4], which is obtained by using a single high-voltage electric pulse to excite the piezoelectric ceramic transducer [5,6]. This technique is simple to implement and can obtain imaging results in detecting geological interfaces and fractures. However, it has limitations in improving the detection distance and imaging resolution and cannot accurately recognize complex geological reflectors. Firstly, the energy of the electric pulse sound source is limited because the maximum voltage at which the transducer can be used is limited [7]; then, the SNR of the reflected wave cannot be further improved, which limits the detection distance of the instrument. On the other hand, the spectrum of the electric pulse sound source is fixed and has a relatively narrow frequency band, and the imaging resolution is insufficient when detecting unconventional reservoirs with complex targets, such as fracture groups and karst caves. Finally, the electric pulse sound source waveform is fixed, and it cannot adapt to the changing borehole formation and physical properties of the reflector.

In order to improve the signal quality, the new generation of acoustic logging instruments use a variety of excitation modes. Ikegami (2003) used a pulsed-signal excitation dipole transducer with an optional main frequency to adapt to different formations [8]. Franco et al. (2006) used pulse and linear frequency modulation (LFM) signals to excite dipoles for different applications [9]. Walker et al. (2015) and Sun et al. (2016) adopted alternative signal excitation methods for monopole and dipole [10,11]. However, most of these new excitation mode techniques are applied to traditional acoustic velocity measurement, and there is no research on excitation waveform design for remote detection imaging. The method of transmitting waveform optimization has been studied extensively in the field of radar [12,13,14,15,16], but it has not been used in acoustic logging. It is important to study the optimal sound source waveform and obtain high-quality reflected wave signals to satisfy the requirements of the remote complex downhole environment.

This paper proposes a high-resolution dipole remote detection method based on the optimal nonlinear frequency modulation (ONLFM) sound source to meet the requirements of long-distance and high-precision remote detection. The ONLFM signal is the frequency modulation signal in which the instantaneous frequency changes nonlinearly with time, and the duration of each frequency can be controlled to adjust the distribution of signal energy at each frequency point [17,18]. Compared with the electric pulse sound source used in traditional technology, it has larger energy and a flexible frequency spectrum that can be adjusted as well. By optimizing the frequency spectrum of ONLFM with the borehole environment information, the bandwidth of the reflected wave can be expanded, and the amplitude of the reflected wave can be enhanced by adjusting the spectrum of the sound source. Thus, the SNR and resolution of the remote detection logging can be improved.

## 2. ONLFM Signal Time Domain Waveform Design

The standard representation of a certain frequency modulation signal can be expressed as [19]:

(1) $$s(t)=a(t)\exp\left[j\varphi (t)\right],$$

where *a*(*t*) is the instantaneous amplitude and *φ*(*t*) is the instantaneous phase.

The Fourier transform of the frequency modulation signal is:

(2) $$S(f)=A(f)\exp\left[j\Phi (f)\right],$$

where *A*(*f*) is the amplitude spectrum and Φ(*f*) is the phase spectrum of the frequency modulation signal.

According to the stationary phase principle [17], the relationship between the group delay and the amplitude spectrum of the FM signal can be obtained as follows:

(3) $$T(f)=\int_{0}^{f}K_{1}{\left|A(v)\right|}^{2}dv+K_{2},$$

where *T*(*f*) is the group delay function. *K*_1_ and *K*_2_ are constants, which can be solved and determined by substituting the boundary conditions *T*(*f*_1_) = 0 and *T*(*f*_2_) = *T*_n_ of Formula (3), where [*f*_1_, *f*_2_] is the frequency band range of the signal and *T*_n_ is the duration of the signal. The instantaneous frequency function, *f*(*t*), can be obtained by the inverse function of *T*(*f*) [20]:

(4) $$f(t)=inv\left\{T(f)\right\}=inv\left\{\int_{0}^{f}K_{1}{\left|A(v)\right|}^{2}dv+K_{2}\right\}.$$

The phase of the frequency modulation signal can be obtained as:

(5) $$\varphi (t)=2\pi \int_{-\infty }^{t}f(\tau )d\tau ,$$

Equations (6) and (7) are substituted into Equation (3) to obtain:

(6) $$s(t)=a(t)\exp\left[2\pi j\int_{0}^{t}inv\left\{\int_{0}^{f}K_{1}{\left|A(v)\right|}^{2}dv+K_{2}\right\}d\tau \right].$$

**Figure 1:**
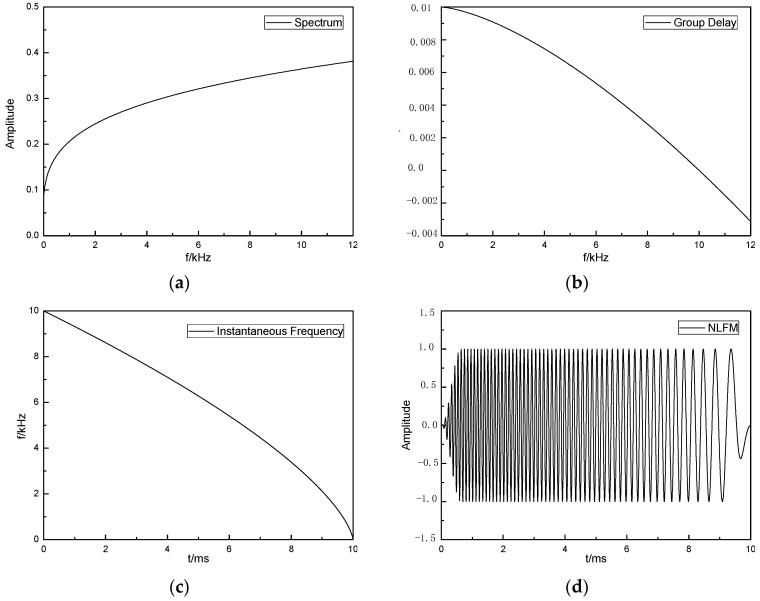
ONLFM signal design, include (**a**) Frequency spectrum, (**b**) Group delay, (**c**) Instantaneous frequency and (**d**) Time domain waveform.

Assuming that the envelope of the transmitted signal is a rectangle, then *a*(*t*) *=* 1 (0 ≤ *t* ≤ *T*_n_), where *T*_n_ is the modulation time of the signal. Ideally, the spectrum of the modulated signal generated according to Equation (6) is consistent with the expected amplitude spectrum, *A*(*f*), as shown in Figure 1. Figure 1a illustrates the frequency spectrum curve, while Figure 1b–d are the instantaneous frequency curve, time domain waveform, and frequency spectrum curve of the ONLFM signal, respectively.

## 3. ONLFM Design Model

It can be seen that the ONLFM signal can be designed by obtaining the frequency spectrum of the desired sound source signal. In this paper, to obtain the optimal frequency spectrum, three models were concerned. First, the optimization model was constructed to obtain the maximum SNR. Second, the maximum resolution was concerned as an objective function. Finally, an optimal model considering both SNR and resolution was constructed to obtain the optimal spectrum of the sound source signal.

### 3.1. Optimization Model Based on Maximum SNR

The reflected wave, *y*(*t*), can be expressed as:

(7) $$y(t)=s_{T}(t)*h_{f}(t)+n_{i}(t)$$

(8) $$Y(f)=S_{T}(f)H(f)+N_{i}(f)$$

where *s*_T_(*t*) is the sound source signal, *h*_f_(*t*) is the target response of the borehole formation reflector, and *n*_i_(*t*) is the stationary white noise, and its power spectral density is:

(9) $$P_{n}(f)=\frac{N_{0}}{2}$$

where *N*_0_ is the noise power density. The instantaneous SNR is the ratio of the instantaneous power of the reflected wave to the average power of the interfering signal. The sound source signal bandwidth is *B*, in which the SNR of the reflected wave signal at time *t*_0_ is:

(10) $$SNR(t_{0})=\frac{2}{N_{0}}{\left|\int_{-B/2}^{B/2}S_{T}(f)H_{f}(f)e^{j2\pi ft_{0}}df\right|}^{2}$$

As can be seen from Equation (10), when the formation target response is *H*_f_(*f*) and the noise power spectral density, *P*_n_(f), is constant, the maximum SNR of the reflected wave changes with the change of the sound source waveform, *S*_T_(*f*). Therefore, the objective function of the maximum SNR can be constructed under the constraint of constant energy:

(11) $$\arg\max SNR=\frac{2}{N_{0}}{\left|\int_{-B/2}^{B/2}S_{T}(f)H_{f}(f)e^{j2\pi ft_{0}}df\right|}^{2}$$

$$s.t.E=\int_{-B/2}^{B/2}{\left|S_{T}(f)\right|}^{2}df$$

where *E* is the power of the sound source signal. According to Schwartz’s inequality:

$$SNR(t_{0})=\frac{2}{N_{0}}{\left|\int_{-B/2}^{B/2}S_{T}(f)H_{f}(f)e^{j2\pi ft_{0}}df\right|}^{2}$$

(12) $$\leq \frac{2}{N_{0}}\int_{-B/2}^{B/2}{\left|S_{T}(f)\right|}^{2}df\int_{-B/2}^{B/2}{\left|H_{f}(f)\right|}^{2}df$$

The equals sign in the above equation can be valid if *S*_T_(*f*) is:

(13) $$S_{T}(f)=H_{f}^{*}(f)e^{-j2\pi ft_{0}}$$

The maximum SNR can be obtained as:

(14) $$SNR_{\max}=\frac{2}{N_{0}}\int_{-B/2}^{B/2}{\left|S_{T}(f)\right|}^{2}df\int_{-B/2}^{B/2}{\left|H_{f}(f)\right|}^{2}df=\frac{2E^{2}}{N_{0}}$$

Therefore, the reflected wave signal can obtain the maximum SNR when *S*_T_(*f*) is the complex conjugate of *H*_f_(*f*).

### 3.2. Optimization Model Based on Maximum Resolution

In the research of radar, the effective correlation bandwidth, *W*_e_, can be used as the evaluation criterion for the imaging resolution performance of the transmitting waveform [21]. The transmitting waveform with the best range resolution performance can be obtained by maximizing *W*_e_. The optimal objective function of *W*_e_ can be established as:

(15) $$\arg\max W_{e}=\frac{{\left|\int_{-B/2}^{B/2}{\left|F(f)\right|}^{2}df\right|}^{2}}{\int_{-B/2}^{B/2}{\left|F(f)\right|}^{4}df}$$

where *F*(*f*) is the reflected wave spectrum. *W*_e_ shows the similarity between the signal distance ambiguity function and the shock function; in other words, the similarity between the signal spectrum and the uniform spectrum. *F*(*f*) can be expressed as:

(16) $$F(f)=S_{T}(f)H_{f}(f)$$

The optimization problem can be expressed as:

(17) $$\arg\max W_{e}=\frac{{\left|\int_{-B/2}^{B/2}{\left|S_{T}(f)H_{f}(f)\right|}^{2}df\right|}^{2}}{\int_{-B/2}^{B/2}{\left|S_{T}(f)H_{f}(f)\right|}^{4}df}$$

$$s.t.E=\int_{-B/2}^{B/2}{\left|S_{T}(f)\right|}^{2}df$$

According to Schwartz’s inequality:

$$W_{e}=\frac{{\left|\int_{-B/2}^{B/2}{\left|S_{T}(f)H_{f}(f)\right|}^{2}df\right|}^{2}}{\int_{-B/2}^{B/2}{\left|S_{T}(f)H_{f}(f)\right|}^{4}df}$$

(18) $$\leq \frac{\int_{-B/2}^{B/2}{\left|S_{T}(f)H_{f}(f)\right|}^{4}df\cdot \int_{-B/2}^{B/2}1^{2}df}{\int_{-B/2}^{B/2}{\left|S_{T}(f)H_{f}(f)\right|}^{4}df}=B$$

The equals sign in the above equation can be valid if *S*_T_(*f*) is:

(19) $$S_{T}(f)=H_{f}^{-1}(f)$$

The maximum value of W_e_ can be obtained as:

(20) $$W_{e\max}=B$$

Therefore, the reflected wave signal can achieve the maximum resolution when *S*_T_(*f*) is the reciprocal of *H*_f_(*f*).

### 3.3. Optimization Model Based on SNR and Resolution

As can be seen from the above analysis, the higher the similarity between |*S*_T_(*f*)| and |*H*_f_(*f*)|, causing the higher SNR, the higher the resolution obtained when the spectral similarity between |*S*_T_(*f*)| and |*H*_f_^−1^(*f*)| is higher. The SNR and resolution are mutually restricted, and the increase of one will cause the decrease of the other, so they cannot reach the maximum at the same time. For remote detection acoustic logging, it is necessary to improve the resolution as far as possible while satisfying a certain SNR. Therefore, this paper constructed a joint optimization model of SNR and resolution. The model can be expressed as maximizing *W*_e_, with energy and minimum SNR constrained:

(21) $$\arg\max W_{e}=\frac{{\left|\int_{-B/2}^{B/2}{\left|S_{T}(f)H_{f}(f)\right|}^{2}df\right|}^{2}}{\int_{-B/2}^{B/2}{\left|S_{T}(f)H_{f}(f)\right|}^{4}df}$$

$$s.t.E=\int_{-B/2}^{B/2}{\left|S_{T}(f)\right|}^{2}df=E_{0},\;SNR=\frac{2}{N_{0}}{\left|\int_{-B/2}^{B/2}S_{T}(f)H_{f}(f)df\right|}^{2}\geq SNR_{0}$$

This is a multi-constraint optimization problem, and it is difficult to obtain the analytical solution. The numerical solution can be obtained by a modern optimization algorithm. The simulated annealing (SA) algorithm is an efficient optimization algorithm, which is a random optimization algorithm based on the Monte Carlo iterative solution strategy [22]. Its principle is based on the similarity between the annealing process of solid matter in physics and general combinatorial optimization problems. Starting from a certain high initial temperature, the global optimal solution of the objective function is randomly found in the solution space with the continuous decline of temperature parameters combined with probabilistic jump characteristics, so that the local optimal solution can jump out probabilistically and eventually tend to the global optimal. In this paper, the SA method is used to solve the optimization model of the sound source waveform. The objective function is constructed as:

(22) $$\arg\min f(S_{T})=-W_{e}(S_{T})$$

The penalty function method is used to transform the multi-constraint problem into an unconstrained problem:

(23) $$\arg\min L(S_{T})=f(S_{T})+m_{k}(c_{1}+c_{2})$$

where:

(24) $$c_{1}={\left(E_{0}-E\right)}^{2}$$

(25) $$c_{2}=\max{\left(0,SNR_{0}-SNR\right)}^{2}$$

where *c*_1_ is the energy constraint condition, *c*_2_ is the SNR constraint condition, and *m*_k_ is the penalty factor. The penalty factor increases with the increase in the number of iterations, forcing the sequence of iteration points of the solution to approach from the outside of the feasible domain to an optimal solution on the boundary of the feasible domain, so that the optimal solution satisfies the constraint conditions. The solving process of the SA algorithm is outlined below [23].

**Figure 2:**
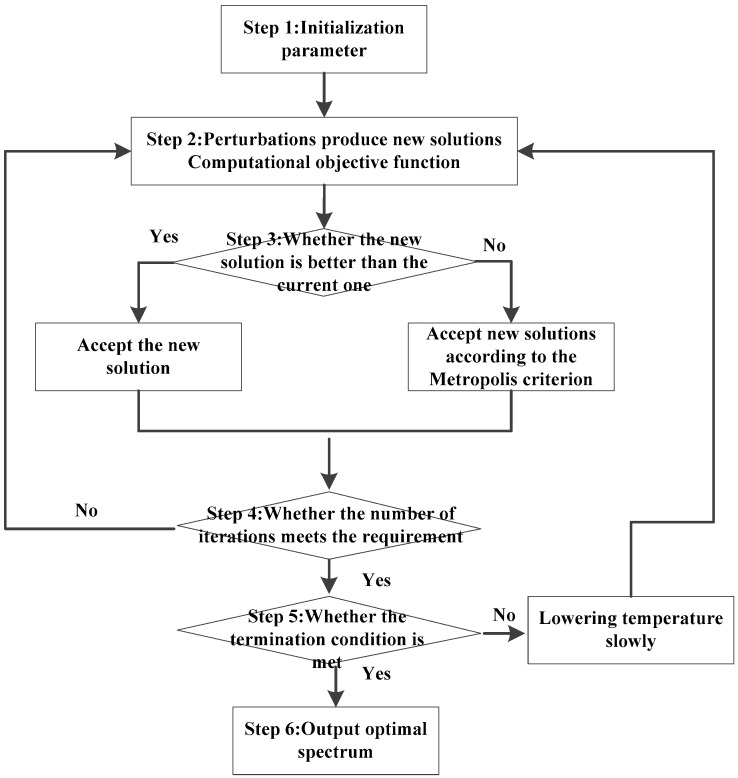
Flowchart of optimizing the NLFM spectrum via the SA method.

The detailed steps of the algorithm are as follows (Figure 2):

Step 1: Parameters required for the initialization algorithm include minimum SNR, *SNR*_0_, noise power spectral density, *N*_0_, signal energy, *E*_0_, formation target response, *H*_f_, initial and end temperature, number of iterations at each temperature, temperature reduction gradient, search space range, Metropolis criterion coefficient, etc.

The whole effective frequency band is divided into *N* equal parts, *S*_T_(*f*_i_) is randomly generated in the search space as the initial spectrum, and the value of the current objective function, *L*(*S*_T_), is calculated.

Step 2: Force random perturbations on each point on the spectrum to obtain a new spectrum and calculate a new objective function value.

Step 3: Compare the two old and new objective function values. If the new objective function value is better than the old value, the new spectrum is accepted as the current optimal spectrum. Otherwise, the new spectrum is accepted as the current optimal spectrum according to the Metropolis criterion.

Step 4: Check whether the number of iterations has been reached. If yes, go to the next step. If no, go to Step 2.

Step 5: Check whether the termination conditions are met. If yes, proceed to the next step. Otherwise, adjust the current temperature according to the temperature gradient and go to Step 2.

Step 6: The globally optimized spectrum is obtained, and the required waveform time domain signal of the sound source is generated according to the stationary phase principle.

After the above steps, the ONLFM sound source waveform signal with maximum resolution can be obtained under the condition of satisfying a certain SNR.

## 4. Numerical Simulation

### 4.1. Method

**Figure 3:**
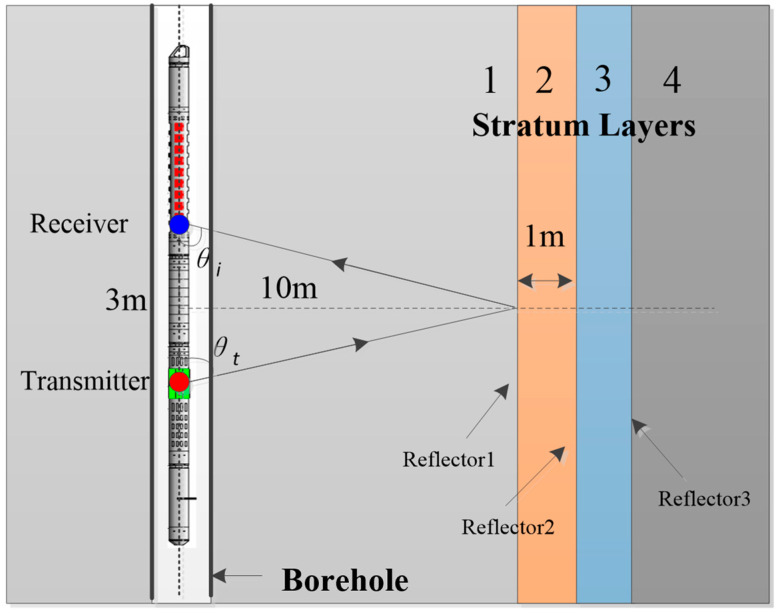
Multi-reflector structure model in borehole formation.

The borehole finite difference model [24,25] was used to simulate the effect of the ONLFM sound source in remote detection logging. It can be seen from the acoustic field characteristics of dipole remote detection that the amplitude–frequency characteristics of the reflected wave were related to the position, inclination, physical properties, reflection coefficient, formation properties, viscoelastic properties, etc. In order to avoid too many variables, making the study impossible, this paper studied the sound source signal optimization under a specific model. The specific model is shown in Figure 3. The borehole radius was 0.1 m, and the source distance between the sound source and the receiving point on the shaft was 3 m. The hard formation environment outside the well was represented by stratum 1. Three graded stratums were set 10 m away from the borehole, which are represented as stratum 2, 3, and 4, respectively. The three stratigraphic interfaces were used as reflectors. The interfacial spacing was 1 m, and the *V*_p_ and *V*_s_ of each layer are shown in Table 1.

**Figure 4:**
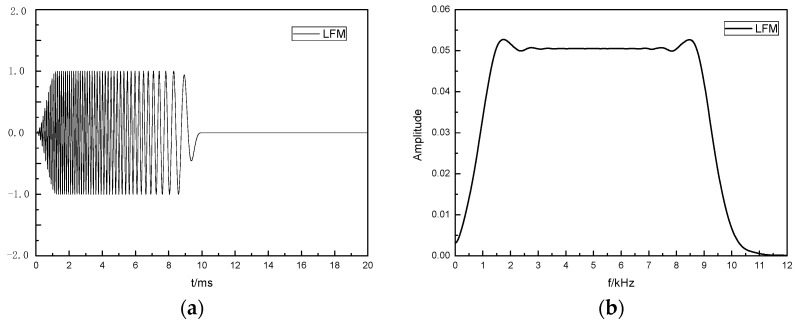
Time domain waveforms and normalized spectrum curves of the LFM sources, include (**a**) Time domain waveform and (**b**) Normalized spectral curve.

First, a wide-band linear frequency modulation (LFM) source was used to estimate the reflector frequency response, *H*_f_(*f*). The LFM signal is a signal whose instantaneous frequency changes linearly with time, and it has a flat and broadband spectral response. The frequency response, *H*_f_(*f*), of the reflector can be estimated according to the frequency spectrum of the reflected wave signal excited by the LFM sound source. The duration of the LFM sound source was 10 ms, the frequency band range was 0.2~10 kHz, and the time domain waveform and spectrum curve are shown in Figure 4.

**Figure 5:**
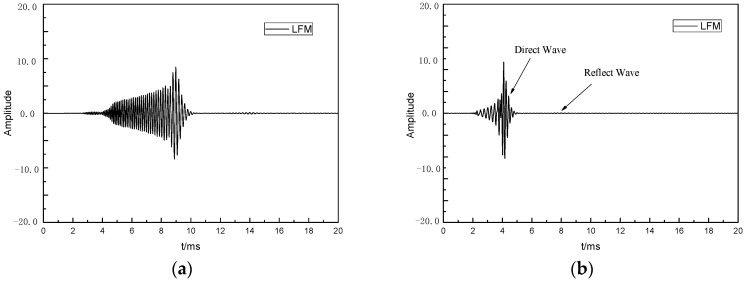
The received waveforms excited by the LFM sound sources, include (**a**) Raw waveform and (**b**) Compressed wave.

The received waveform obtained via excitation of the LFM sound source is shown in Figure 5a. Since the duration of the LFM excitation signal was longer, the duration of the excitation direct wave was longer, and the reflected wave was submerged in the direct wave, pulse compression processing was required for the received waveform. The basic principle of pulse compression is the matching filter technology [21]. In this paper, the complex conjugate signal of the transmitted signal was used as the matching filter, and the received signal was convolved with the matching filter to obtain the compressed output signal. The received waveform after processing is shown in Figure 5b.

**Figure 6:**
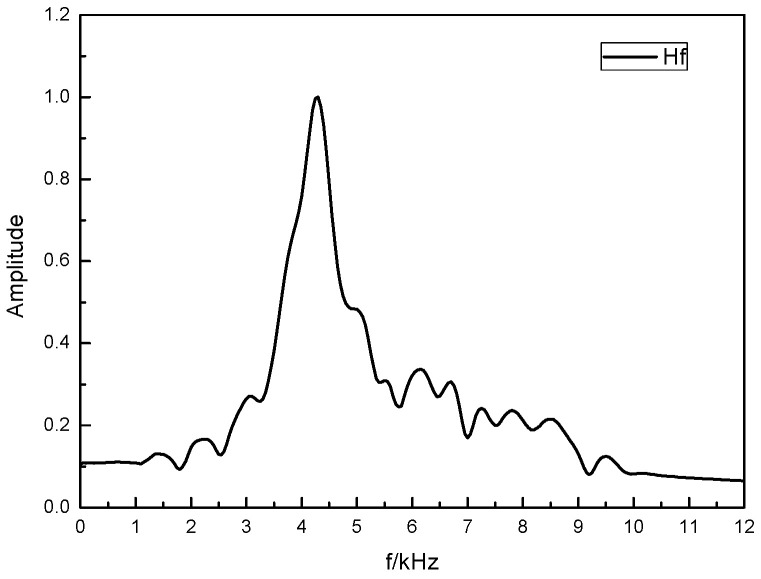
Reflector frequency response curve.

The reflector frequency response curve, *H*_f_(*f*), can be obtained by extracting the reflected wave spectrum in the received waveform, as shown in Figure 5b, dividing it by the sound source spectrum, as shown in Figure 4, and performing the root operation, as shown in Figure 6.

**Figure 7:**
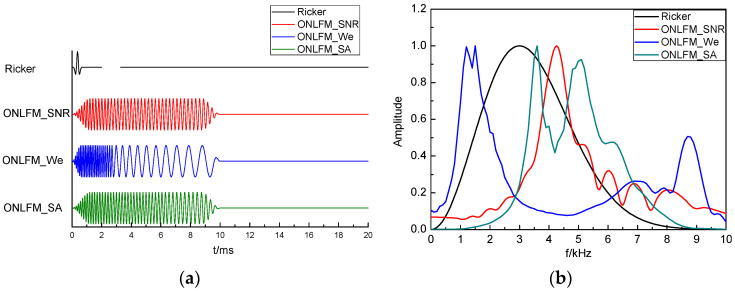
Time domain waveforms and normalized spectrum curves of the four sound sources, include (**a**) Time domain waveform and (**b**) Normalized spectral curve.

The next step was to verify the detection effect of the ONLFM sound source on the reflector group. Four waveforms of sound sources were used for sound field simulation: the traditional Ricker wavelet, which is expressed as Ricker; the optimized waveform with maximum SNR, which is expressed as ONLFM_SNR; the optimized waveform with maximum resolution, which is expressed as ONLFM_We; the optimized waveform with both SNR and resolution, which is expressed as ONLFM_SA. The main frequency of the Riker wavelet was 3 kHz, and the duration of the three ONLFM signals was 10 ms. The time domain waveforms and normalized spectrum curves of the four sound sources are shown in Figure 7.

### 4.2. Results

**Figure 8:**
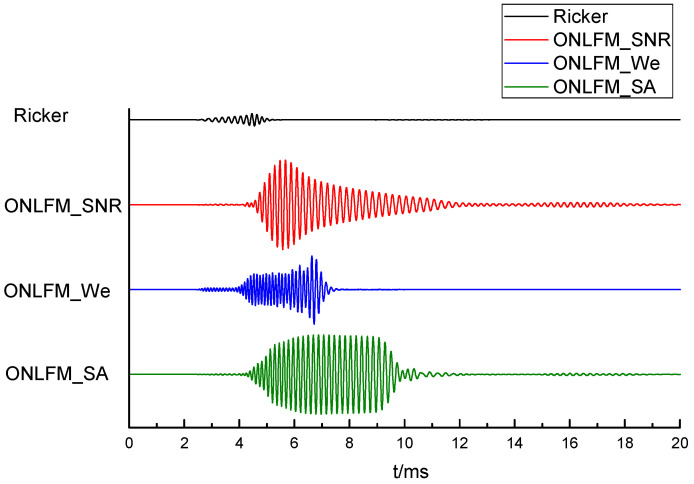
The received waveforms excited by four kinds of sound sources.

**Figure 9:**
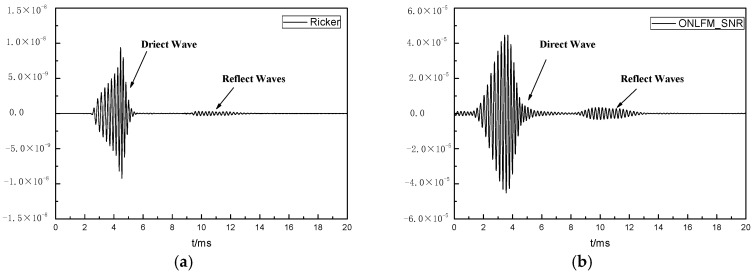

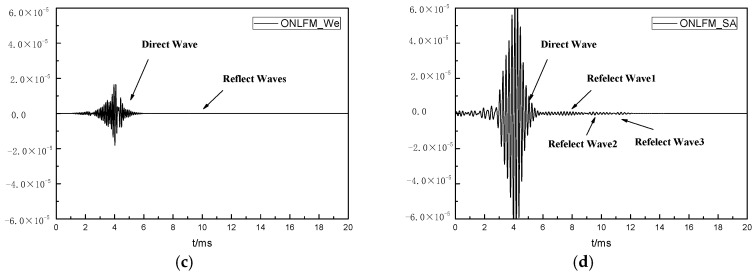
The received waveforms after pulse compression, include (**a**) Ricker, (**b**) ONLFM_SNR, (**c**) ONLFM_We and (**d**) ONLFM_SA.

The received waveforms excited by four kinds of sound sources are shown in Figure 8. It can be seen that the direct wave excited by three kinds of ONLFM signals had a long duration, and the reflected wave was submerged in the direct wave, so it was necessary to pulse compress the received waveforms. The received waveforms after pulse compression are shown in Figure 9.

**Figure 10:**
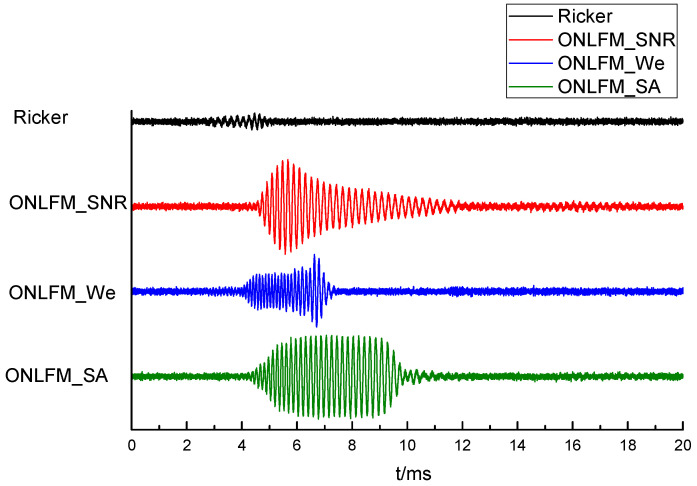
Received waveforms in the time domain after loading noise.

**Figure 11:**
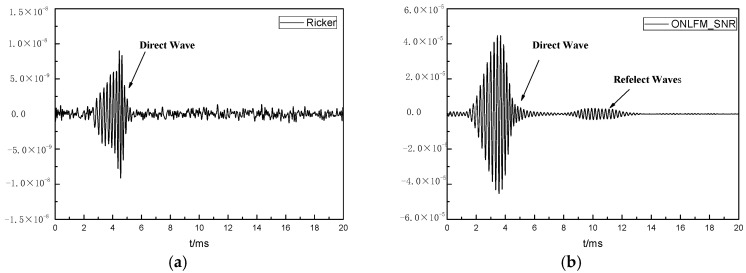

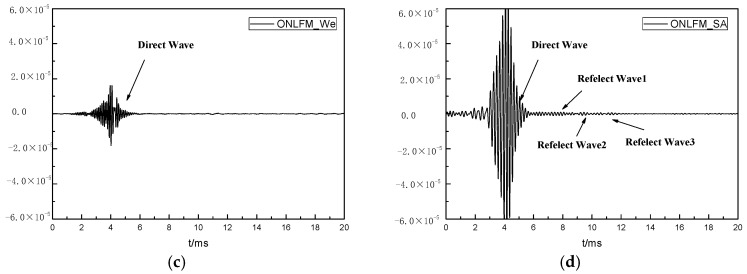
The received waveforms after data processing, include (**a**) Ricker, (**b**) ONLFM_SNR, (**c**) ONLFM_We and (**d**) ONLFM_SA.

Next, we considered the situation when the SNR of the received waveform was low. White noise of the same magnitude was loaded on the four received waveforms in Figure 5, as shown in Figure 10. To process the waveform data, bandpass filtering was performed on the Ricker received waveforms, and pulse compression was performed on the three ONLFM received waveforms, as shown in Figure 11.

### 4.3. Discussion

The four kinds of sound sources we used have different characteristics. The spectrum of the Riker wavelet is independent of the environment and the signal energy is minimal. The three ONLFM signals have strong signal energy and different spectral characteristics, which is related to the environment: the spectrum of ONLFM_SNR is the same as that of the reflector frequency response, *H*_f_(*f*), the spectrum of ONLFM_We is reciprocal to that of *H*_f_(*f*), and the spectrum of ONLFM_SA is in between the above two.

As can be seen from the Figure 9, reflected wave groups could be identified in the received waveforms stimulated by Ricker, but multiple reflected waves were mixed together, unable to distinguish a single reflector, and the reflected wave amplitude was small. ONLFM_SNR excited the reflection wave with the largest amplitude among the four curves, but also could not distinguish a single reflector. ONLFM_We excited the reflection wave and could distinguish three kinds of reflectors, but the amplitude of the reflection wave was the smallest among the four kinds of curves, and it could not be identified in the curve of the same scale. ONLFM_SA excited the reflection wave and had sufficient amplitude and resolution to clearly identify the reflection wave of three reflectors in the curve.

When the noise was considered, it can be seen from Figure 11 that the reflected waves excited by Ricker and ONLFM_We were submerged in noise and could not be identified because of the small amplitude. The received waveform of ONLFM_SNR could recognize the reflection wave group but still could not distinguish the single reflector. The received waveform stimulated by ONLFM_SA could still recognize three reflectors under the condition of large noise, which met the requirements of SNR and resolution for remote detection at the same time.

## 5. Conclusions

In this paper, an ONLFM sound source signal was studied, which could provide the highest resolution of the reflected wave, satisfying a certain SNR, so as to meet the needs of long-distance and high-precision imaging for dipole shear wave remote detection. The ONLFM sound source signal model was derived to achieve maximum SNR, maximum resolution, and optimal SNR and resolution, respectively. The optimized model is a multi-constraint optimization problem, and the simulated annealing method was used to solve it.

Then, the responses of four sound sources: Riker wavelet, ONLFM with maximum SNR, ONLFM with maximum resolution, and ONLFM with both SNR and resolution, were simulated, respectively, using the finite difference method in the multi-target formation borehole model, and the recognition effects of the received waveforms under the condition of low SNR were compared. The results showed that the reflection waves excited by the Riker wavelet and the ONLFM with maximum resolution were submerged in the noise and could not be identified. The received waveform of the ONLFM with maximum SNR could recognize the reflected wave group but could not distinguish the single reflector. The received waveform generated by the ONLFM with both SNR and resolution could recognize three reflectors in the case of large noise, which met the requirements of SNR and resolution for remote detection at the same time.

## Figures and Tables

**Table 1:** The *V*_p_ and *V*_s_ of each stratum layer in the model.

Layer	*V*_p_ (m/s)	*V*_s_ (m/s)
1	4000	2300
2	3800	2100
3	3700	2000
4	3600	1900

## Data Availability

The raw data supporting the conclusions of this article will be made available by the authors on request.
